# *Anopheles funestus* Populations across Africa Are Broadly Susceptible to Neonicotinoids but with Signals of Possible Cross-Resistance from the GSTe2 Gene

**DOI:** 10.3390/tropicalmed8050244

**Published:** 2023-04-24

**Authors:** Tatiane Assatse, Magellan Tchouakui, Leon Mugenzi, Benjamin Menze, Daniel Nguiffo-Nguete, Williams Tchapga, Sevilor Kekeunou, Charles S. Wondji

**Affiliations:** 1 Centre for Research in Infectious Diseases (CRID), Yaoundé P.O. Box 13501, Cameroon; magellan.tchouakui@crid-cam.net (M.T.); leon.mugenzi@crid-cam.net (L.M.); benjaminmenze@crid-cam.net (B.M.); daniel.nguiffo@crid-cam.net (D.N.-N.); williams.tchapga@crid-cam.net (W.T.); 2 Parasitology and Ecology Laboratory, Department of Animal Biology and Physiology, Faculty of Science, University of Yaoundé 1, Yaoundé P.O. Box 812, Cameroon; skekeunou@gmail.com; 3 Department of Vector Biology, Liverpool School of Tropical Medicine, Pembroke Place, Liverpool L3 5QA, UK; 4 International Institute of Tropical Agriculture (IITA), Yaoundé P.O. Box 2008, Cameroon

**Keywords:** malaria, *Anopheles funestus*, neonicotinoids, clothianidin, L119F-GSTe2, MERO

## Abstract

Evaluating the susceptibility of malaria vectors to the new WHO-recommended products is a key step before large-scale deployment. We mapped the susceptibility profile of *Anopheles funestus* to neonicotinoids across Africa and established the diagnostic doses of acetamiprid and imidacloprid with acetone + MERO as solvent. Indoor resting *An. funestus* were collected in 2021 in Cameroon, Malawi, Ghana and Uganda. Susceptibility to clothianidin, imidacloprid and acetamiprid was evaluated using CDC bottle assays and offsprings of the field-caught adults. The L119F-GSTe2 marker was genotyped to assess the potential cross-resistance between clothianidin and this DDT/pyrethroid-resistant marker. Mosquitoes were susceptible to the three neonicotinoids diluted in acetone + MERO, whereas low mortality was noticed with ethanol or acetone alone. The doses of 6 µg/mL and 4 µg/mL were established as diagnostic concentrations of imidacloprid and acetamiprid, respectively, with acetone + MERO. Pre-exposure to synergists significantly restored the susceptibility to clothianidin. A positive correlation was observed between L119F-GSTe2 mutation and clothianidin resistance with the homozygote resistant mosquitoes being more able to survive than heterozygote or susceptible. This study revealed that *An. funestus* populations across Africa are susceptible to neonicotinoids, and as such, this insecticide class could be effectively implemented to control this species using IRS. However, potential cross-resistance conferred by GSTe2 calls for regular resistance monitoring in the field.

## 1. Introduction

Malaria remains a major public health problem in sub-Saharan Africa. The malaria cases increased from 245 million in 2020 to 247 million in 2021; most of that increase came from the WHO African Region which accounts for 95% of global cases [1]. Although significant gains in malaria control have been achieved since 2000, insecticide resistance has emerged as one of the major obstacles to the global fight against malaria. Malaria vectors have become resistant to all four insecticide classes traditionally used in vector control: pyrethroids, organophosphates, organochlorines and carbamates [2]. Resistance to pyrethroids is of particular concern because they are widely used in indoor residual spraying (IRS) and are the main insecticide class approved for impregnating long-lasting insecticidal nets (LLINs). The continued spread of such resistance could threaten the malaria control progress achieved, leading to operational failures of prevailing control measures [3]. Regarding this challenge, the current recommendations for insecticide resistance management rely on the tactical deployment of the active ingredients used for IRS and on LLINs in rotation, combinations (particularly LLINs), mosaics and mixtures [4,5]. Although these strategies have been established, countries have generally not been able to put insecticide rotation into practice to manage insecticide resistance due to the limited available class of insecticides and the long time required to develop new molecules. For four decades, pyrethroids were the remaining chemical class recommended to the public health market and have proved to be highly successful both for LLINs and IRS [6,7]. However, widespread resistance to pyrethroids has led to the urgent need for new active ingredient insecticides with neonicotinoids and pyrroles that were recently pre-qualified by WHO [3].

Neonicotinoids are active substances used in plant protection products to control harmful insects [8] mainly due to their water solubility, which allows them to be applied to soil and taken up by plants. This class includes seven insecticides among which clothianidin has low mammalian toxicity and is primarily used against piercing–sucking insects of major crops [9,10]. Besides clothianidin, we have acetamiprid, imidacloprid and thiamethoxam which are prominently used in agriculture. These insecticides target the nicotinic acetylcholine receptor (nAChR) in the insect’s central nervous system [9,10]. Each insecticide from this group showed at the molecular level differential activity against the nAChR protein subunit of *Anopheles gambiae*, indicating that they probably have differential efficacies on insects [9]. Clothianidin induced the highest mortality in *Culex quinquefasciatus* [11] compared to other insecticides of the group. Against *Aèdes. aegypti* and *A*n. *gambiae*, six neonicotinoids tested had poor individual efficacies but induced higher levels of insecticidal action when in combination with the synergist PBO (Pyperonyl ButOxide), showing an implication of P450s in mosquito’s ability to withstand exposure to this insecticide class [12].

New IRS formulations, SumiShield™ 50WG (water-dispersible granules containing clothianidin as the active ingredient, which is applied in IRS at a target dose of 300 mg ai/m^2^) (Sumitomo chemical Co., Ltd., Tokyo, Japan) and Fludora Fusion™ WP-SB (wettable powder in a water-soluble bag containing a mixture of clothianidin and deltamethrin (pyrethroid), applied at 225 mg ai/m^2^) (Bayer Crop science, Monheim am Rhein, Germany), became WHO pre-qualified vector control products in 2017 and 2018, respectively. Recently, a study on *An*. *gambiae* revealed a reduced susceptibility of some field populations of *An. gambiae* to highlight the need to monitor such susceptibility patterns to this new insecticide in other major malaria vectors such as *An. funestus* and more so across different African regions. It was also shown that using acetone or ethanol alone as a solvent for clothianidin can overestimate resistance levels in mosquitoes due to the crystallisation issue [13]. But the addition of MERO (rapeseed oil that prevents the crystallization of the insecticide) was demonstrated to prevent the crystallisation and significantly increase the efficacy of this clothianidin [13]. This highlights the urgent need to establish the diagnostic dose of other neonicotinoids to monitor the development of resistance to this class of insecticide. Here, we evaluated the susceptibility profile of *An. funestus* populations to three neonicotinoids (clothianidin, imidacloprid and acetamiprid) across Africa using three different solvents and established the diagnostic dose of acetamiprid and imidacloprid with acetone+MERO as solvent. Additional assays were performed with the synergists piperonyl butoxide (PBO), di-ethyl Maleate (DEM) and s,s,s–tri-butylphosphorotrithioate (DEF) known to knockdown, respectively, the family of Cytochrome P450 enzymes, the GSTes enzymes family and the esterases enzymes family; responsible for the metabolic resistance in mosquitoes.

This study revealed that *An. funestus* populations across Africa are also susceptible to neonicotinoids but with possible cross-resistance from the *GSTe2* gene.

## 2. Materials and Methods

### 2.1. Study Sites and Mosquitoes Sampling

**Figure 1:**
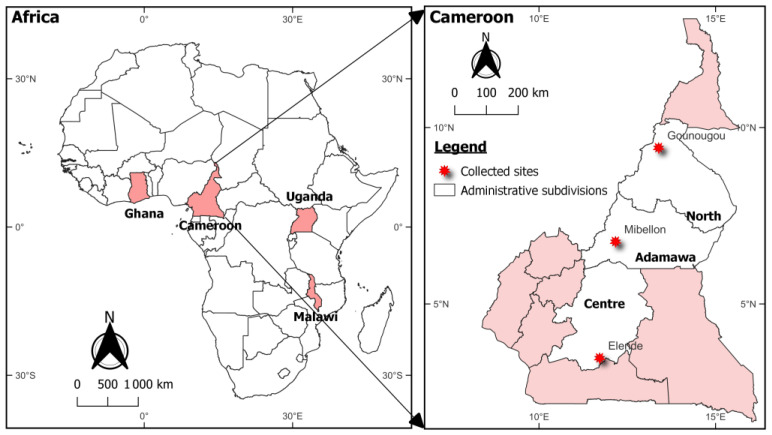
Geographical representation of the collection sites.

Mosquitoes were collected in agricultural settings in four countries from the African regions (Figure 1). In Cameroon, mosquitoes were collected from February to December 2021 (considering the four seasons of the tropical regions) at Elende in the central region, Mibellon in the Adamawa region and Gounougou in the north region. Mosquitoes were sampled in October 2021 in Mayuge (0°23′10.8″ N, 33°37′16.5″ E), located in the eastern part of Uganda. In Ghana, sample collection was carried in August 2021 in Atatem (5°56′ N, 1°37′ W) in the Adansi Asokwa District of the Ashanti Region. In Malawi, mosquitoes were sampled in June 2021 in Chikwawa district (16°1′ S; 34°47′ E) in the southern part of the country. Mosquitoes were sampled at the adult stage (indoor resting female) using an electric aspirator. At least 1000 indoor-resting females were collected from each location. They were maintained for 4 to 5 days to allow them to reach the stage fully and were checked daily for survival. The gravid mosquitoes were then gently and individually introduced into 1.5 mL Eppendorf tubes containing a 1 square cm filter paper inserted into the bottom of the tube as described by Morgan and collaborators [14]. The filter paper was moistened and excess water removed. The cap of the 1.5 mL tube was pierced with 3 holes to allow air into the tube. The tubes were checked daily for the presence of eggs. Females that laid eggs were carefully removed from the tubes and transferred into 1.5 mL tubes with silica gel for further molecular analysis. Eggs were allowed to hatch in a small cup and later moved to larvae bowls for rearing. Larvae were reared in mineral (bottled) and fed with Tetramin^TM^ (Chewy, FL, USA) baby fish food every day. The water of each larvae bowl was changed every two days to reduce the mortality. The F1 adults generated were randomly mixed in cages for subsequent bioassays with neonicotinoids. Two to five-day-old female mosquitoes (F1) from the collected adults (F0) were used for the bioassays as well as the resistant lab strain FUMOZ.

### 2.2. Molecular Identification of Species Collected

Mosquitoes were morphologically identified according to the Gillies and De Meillon keys [15] as belonging to *An. funestus* group. Genomic DNA was then extracted from a subset of mosquitoes using the Livak protocol [16] and *An. funestus* members were differentiated using species-specific PCR performed according to the protocol of Koekemoer et al. [17].

### 2.3. Determination of Susceptibility Profile to Neonicotinoids and Establishment of the Diagnostic Dose of Imidacloprid and Acetamiprid Diluted in Acetone + MERO

Bioassays were conducted with emerged females of 2 to 5 days old (4–5 replicates per insecticide) using neonicotinoids, including three chemical compounds: clothianidin, imidacloprid and acetamiprid. They were technical materials from Sigma (PESTANAL^®^, analytical standard, Sigma-Aldrich, Dorset, UK). These insecticides were diluted in absolute ethanol or acetone alone at the diagnostic doses of 150 µg/mL, 200 µg/mL and 75 µg/mL for clothianidin, imidacloprid and acetamiprid, respectively. When the MERO^®^ (Bayer Crop science, Monheim am Rhein, Germany) combined with acetone was used as a solvent to assess the susceptibility profile of mosquitoes from the different study sites, the diagnostic doses were unchanged for imidacloprid and acetamiprid; however, for clothianidin, 90 µg/mL was used as previously recommended by Bayer and used by previous authors [13,18]. Furthermore, different insecticide concentrations were tested using the susceptible lab strain Kisumu to evaluate the diagnostic doses of imidacloprid and acetamiprid with acetone + MERO as solvent. For imidacloprid, we used concentrations ranging from 1, 2.5, 3, 4, 5, 10, 30, 50 and 200 µg/mL and for acetamiprid, we used concentrations ranging from 1, 2, 3, 5, 10, 20, 40 and 75 µg/mL. The stock solution of the acetone/MERO^®^ mixture was made by pipetting 89 µL of MERO^®^ and adding to 100 mL of acetone according to WHO protocol [19]. The 250 mL CDC bottles used were coated with one (1) mL of each mixture (insecticide + solvent for tests and solvent alone for controls) for a single insecticide.

The procedure consisted of putting 15 to 25 females (2–5 days old) in four pre-coated insecticide bottles and then exposing them for one hour. Control mosquitoes were exposed in bottles coated with the solvent alone. After exposure, mosquitoes were transferred into cleaned paper cups for observation, and the knockdown was reported. Mortality was therefore monitored and reported every 24 h until seven days to better capture the effect of insecticides on the mosquitoes since the latter are known to be slow acting.

### 2.4. Synergist Assays

In addition to the standard test, complementary assays associating the synergists piperonyl butoxide (PBO), di-ethyl Maleate (DEM) and s,s,s–tri-butylphosphorotrithioate (DEF) to clothianidin were performed to assess the role of metabolic enzymes in cases of reduced susceptibility. Mosquitoes were pre-exposed for one hour to synergists (4% PBO, 8% DEM or 0.25% DEF) before being exposed to the insecticide for one hour more. The mortality was also followed up until 7 days, and the Chi-square test was used to compare the mortality between this assay and those without synergists.

#### Potential Cross-Resistance between Neonicotinoids and Pyrethroids

The potential cross-resistance between neonicotinoids and pyrethroids was assessed with field mosquitoes from Elende exposed to clothianidin 0.25 µg/mL diluted in acetone + MERO for 20 min. Alive and dead mosquitoes from the bioassays were used for genotyping the L119F-GTSe2 metabolic resistance marker conferring DDT/pyrethroids resistance in *An. funestus* mosquitoes [20]. To do this, PCR reactions (15 µL) contained 1 µL of genomic DNA, 1.5 µL of 10× buffer A, 0.75 µL of 25 mM MgCl_2_, 0.12 µL of 25 mM dNTPs and Kapa Taq, 0.51 µL of each primer and 10.49 µL of sigma water [21]. Samples were run in a thermocycler (Bulldog Bio, Inc., Portsmouth, NH, USA) with temperature cycling conditions of 5 min at 95 °C followed by 30 cycles of 94 °C for 30 s, 58 °C for 30 s and 72 °C 1 min and then 72 °C for 10 min.

To establish the statistical significance of any association between the *GSTe2* marker and the ability of mosquitoes to survive clothianidin exposure, we used the odds ratio and Fisher exact test.

## 3. Results

### 3.1. Susceptibility Profile of Mosquitoes to Clothianidin

**Figure 2:**
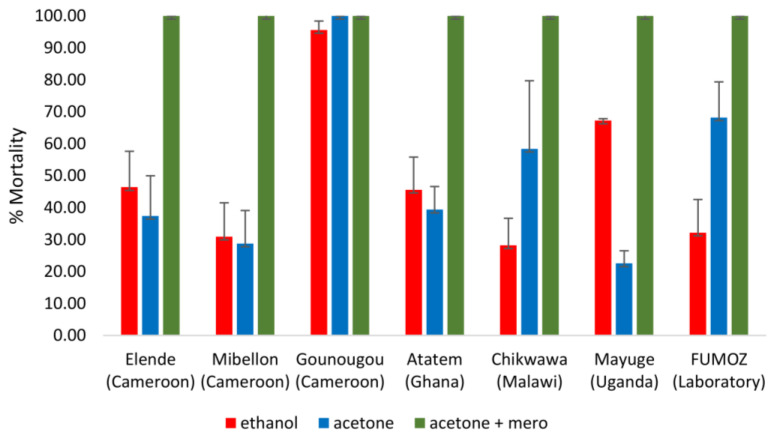
Susceptibility profile of *An. funestus* s.l. populations to clothianidin across Africa with 150 µg/mL concentrations when diluted in ethanol or acetone alone but 90 µg/mL when diluted in acetone + MERO (89 ppm). The mortality rate of mosquitoes from different sites 7 days post-exposure to clothianidin. Results are the average of percentage mortalities from four to five replicates each. The bars represent the standard error on the mean (SEM).

The cocktail PCR revealed that most mosquitoes (more than 90%) from Elende, Mibellon, Gounougou, Ghana, Malawi and Uganda belonged to *An. funestus* s.s. However, the results of bioassays consider that all the mosquitoes collected, whatever the specific species (*An. funestus* s.l.). The susceptibility profile of mosquitoes to clothianidin was solvent-dependent in most of the locations. When combining the MERO with acetone and using it as a solvent, the mosquitoes were susceptible to clothianidin whatever the site. However, lower mortality was observed when the insecticide was diluted in either ethanol or acetone alone (Figure 2) except in Gounougou where we observed a susceptibility of mosquitoes to clothianidin diluted in all the solvents. When using acetone alone as a solvent, the mortality varied from 22.55% ± 3.95 in Mayuge, 28.77% ± 10.34 in Mibellon, 37.42% in Elende, 39.38% ± 7.23 in Atatem (Ghana), 58.43% ± 21.29 in Chikwawa (Malawi), to 100% in Gounougou. When dissolved in ethanol, the susceptibility of mosquitoes to clothianidin varied from 28.21% ± 8.46 Chikhwawa (Malawi) 30.92% ± 10.60 in Mibellon, 45.59% ± 10.23 in Atatem (Ghana), 46.40% ± 11.25 in Elende, 67.25% ± 0.58 in Mayuge to 95.59% ± 2.82 in Gounougou (Figure 1). When using acetone + MERO as solvent, we observed a full susceptibility of mosquitoes to clothianidin in all the locations tested. The same pattern was observed with the resistant lab strain Fumoz which showed high mortality to clothianidin diluted in either ethanol or acetone alone but a full susceptibility when diluted in acetone+MERO. However, there was no mortality in control tubes.

### 3.2. Susceptibility Profile to Clothianidin with Synergists

**Figure 3:**
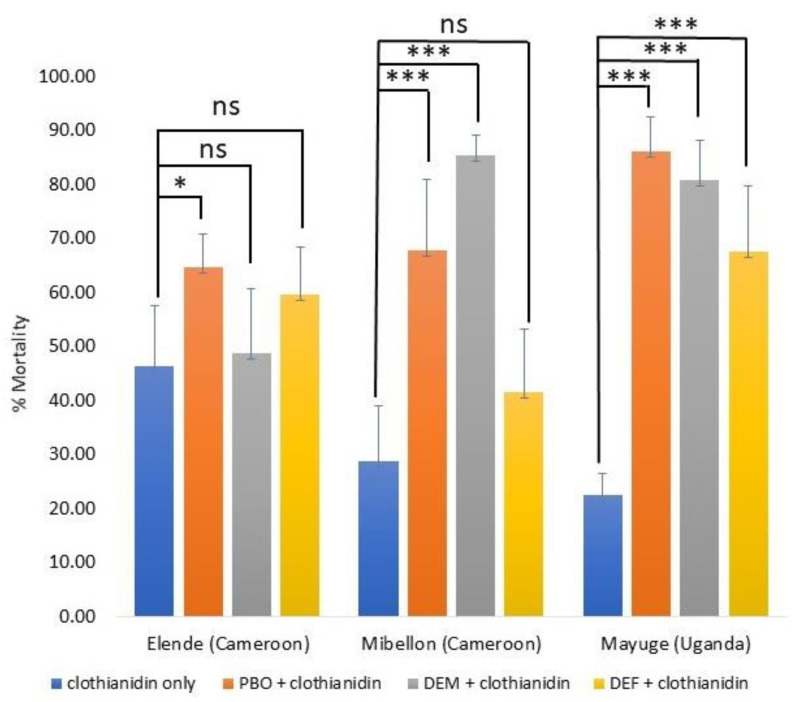
Susceptibility profile of *An. funestus* s.l. to clothianidin with synergists. Effect of pre-exposure to synergist PBO, DEM and DEF against clothianidin 150 µg/mL diluted in acetone on *An. funestus* from Elende, Mibellon and Mayuge. Results are the average of percentage mortalities from four to five replicates each. The bars represent the standard error on the mean (SEM). The stars (*) represent the significance level. ns means non significant.

Synergist assays performed with PBO, DEM and DEF showed a significant recovery of the susceptibility of mosquitoes from Elende, Mibellon and Mayuge to clothianidin diluted in either ethanol or acetone alone (Figure 3). In An. funestus from Elende, the mortality increased from 46.40 ± 11.25 for clothianidin alone to 64.71 ± 6.21 for PBO + clothianidin (χ^2^ = 4.138; *p* = 0.0419), to 48.77 ± 12.05 for DEM + clothianidin (χ^2^ = 0.068; *p* = 0.7936) and 59.59 ± 8.90 for DEF + clothianidin (χ^2^= 2.043; *p* = 0.1529). In Mibellon, the mortality rate moved from 28.77 ± 10.34 for clothianidin only (diluted in acetone) to 67.83 ± 13.02 for PBO + clothianidin (χ^2^ = 19.554; *p* < 0.0001), 85.45 ± 3.67 for DEM + clothianidin (χ^2^ = 43.428; *p* < 0.0001) and 41.47 ± 11.85 for DEF + clothianidin (χ^2^ = 2.266; *p* = 0.1322). The same restoration of susceptibility was observed in Mayuge, with the mortality rate moving from 22.55 ± 3.95 for clothianidin only to 86.17 ± 6.47 for PBO + clothianidin (χ^2^ = 52.053; *p* < 0.0001), 80.82 ± 7.26 for DEM + clothianidin (χ^2^ = 41.971; *p* < 0.0001) and 67.58 ± 12.22 for DEF + clothianidin (χ^2^ = 25.039; *p* < 0.0001).

### 3.3. Susceptibility Profile of Mosquitoes to Imidacloprid

**Figure 4:**
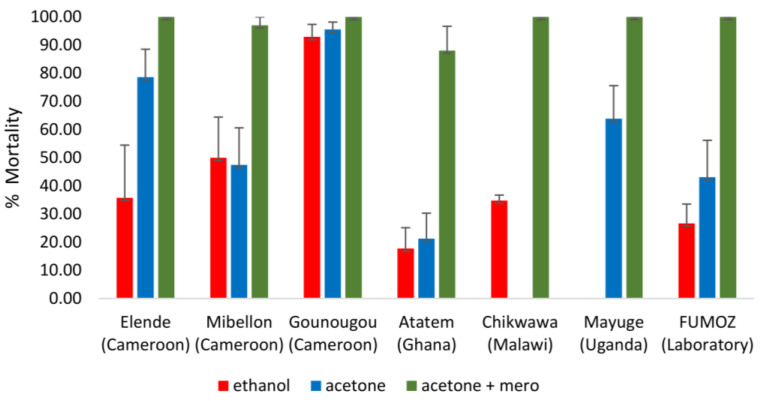
Susceptibility profile of *An. funestus* s.l. to imidaclopridacross Africa. The mortality rate of mosquitoes from different sites 7 days post-exposure to imidacloprid 200 µg/mL dissolved in various solvents compared to pyrethroid resistant strain FUMOZ. Results are the average of percentage mortalities from four to five replicates each. The bars represent the standard error on the mean (SEM); CMR: Cameroon; lab: laboratory.

As observed with clothianidin, low mortality was recorded for mosquitoes exposed to imidacloprid diluted in either ethanol or acetone, but had higher mortality when compared to imidacloprid diluted in acetone + MERO (Figure 4). The mortality rate with imidacloprid diluted in absolute ethanol ranged from 17.74% ± 7.41 in Atatem, 34.77% ± 1.96 in Malawi, 35.77% ± 18.70 at Elende, 49.99% ± 14.44 in Mibellon to 92.95% ± 4.40 in Gounougou, showing that *An. funestus* from Atatem (Ghana) are the less susceptible to imidacloprid. When using acetone alone as solvent, the mortality ranged from 21.28% ± 9.03 in Ghana, 47.46% ± 13.16 in Mibellon, 63.82% ± 11.78 in Uganda, 78.65% ± 9.88 at Elende, to 95.58% ± 4.40 in Gounougou. Mosquitoes were fully susceptible in almost all the localities except those of Ghana (88.10% ± 8.58 mortality) and Mibellon (92.95% ± 2.94 of mortality). The pyrethroid-resistant strain, FUMOZ, showed the same pattern with 26.70% ± 6.84, 43.09% ± 13.08 and 100% of mortality to imidacloprid diluted in ethanol, acetone and acetone + MERO respectively. In contrast, no mortality was recorded in the control group.

### 3.4. Susceptibility Profile of Mosquitoes to Acetamiprid

**Figure 5:**
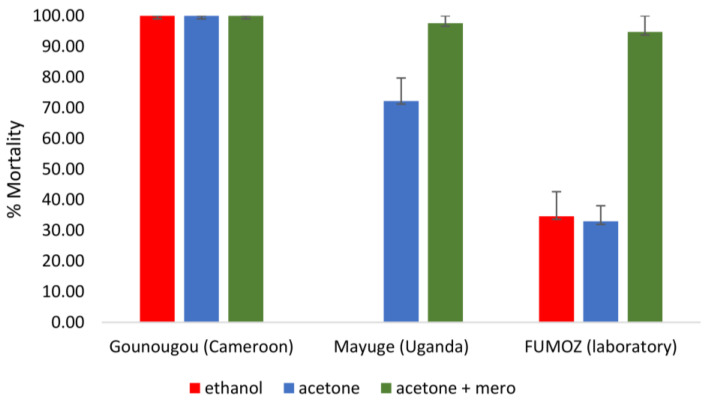
Susceptibility profile of *An. funestus* s.l. to acetamiprid across Africa. The mortality rate of mosquitoes from different sites 7 days post-exposure to acetamiprid 75 µg/ mL dissolved in various solvents compared to pyrethroid-resistant strain FUMOZ. Results are the average of percentage mortalities from four to five replicates each. The bars represent the standard error on the mean (SEM); CMR: Cameroon; lab: laboratory.

The susceptibility to acetamiprid was evaluated with mosquitoes from Gounougou (Cameroon) and Mayuge (Uganda) (Figure 5). Mosquitoes from Gounougou were fully susceptible to acetamiprid, whatever the solvent. The mortality with mosquitoes from Uganda was 72.21% ± 7.50 for acetamiprid diluted in acetone alone compared to 97.62% ± 2.38 with acetone + MERO. The resistant lab strain FUMOZ showed a mortality of 34.62% ± 8.00, 32.97% ± 5.07 and 94.74 ± 5.26 toacetamiprid diluted in ethanol, acetone and acetone + MERO, respectively, with no mortality in controls.

### 3.5. Establishment of Diagnostic Dose of Imidacloprid and Acetamiprid Using Acetone and Mero as Solvent

**Figure 6:**
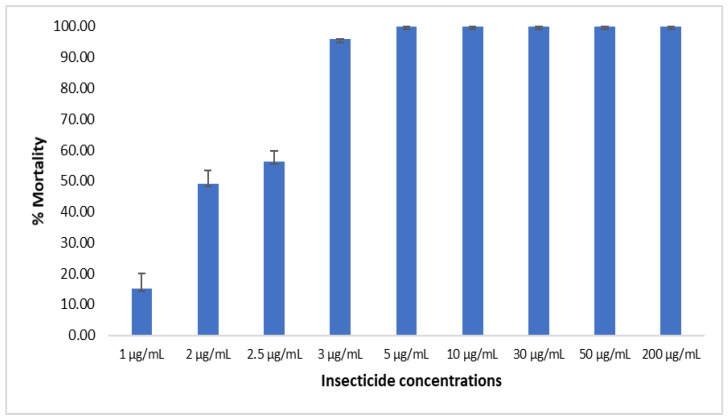
Assessment of diagnostic concentration of imidacloprid using acetone + MERO. Mortality rate 24 h post-exposure to eight different insecticide concentrations and using acetone + MERO as solvent. Results are the average of percentage mortalities from four-five replicates each. The bars represent the standard error on the mean (SEM).

Due to the very high mortality observed with imidacloprid and acetamiprid diluted in acetone + MERO, we decided to establish the diagnostic concentration of both insecticides using the susceptible lab strain KISUMU following the previously established protocol [12]. For imidacloprid, 24 h after exposure, the recorded percentage of mortality ranged from 15.14% ± 4.9 for 1 µg/mL, 49.17% ± 4.2 for 2 µg/mL, 56.34% ± 3.51 for 2.5 µg/mL to 95.82% ± 0.13 for 3 µg/mL and 100% for 5 µg/mL and above (Figure 6). As the concentration of 3 µg/mL induced mortality, ˃90% at 60 min of exposure on the susceptible lab strain Kisumu, 6 µg/mL could the suitable concentration for assessing the susceptibility profile of field population of malaria vectors to imidacloprid when using acetone + MERO based on WHO 2022 criteria to define a diagnostic dose of insecticide [19].

**Figure 7:**
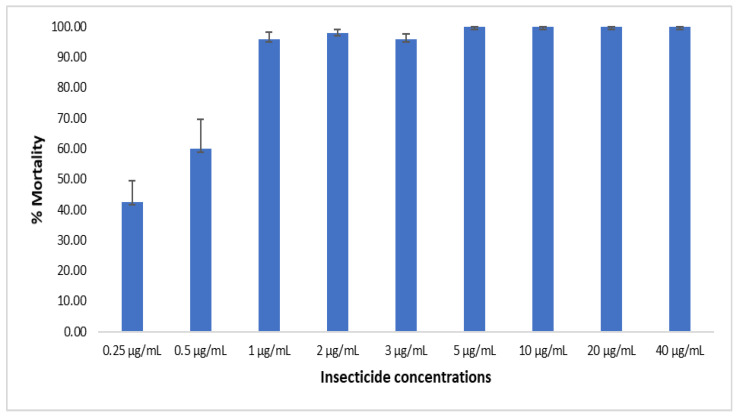
Assessment of diagnostic concentration of acetamiprid using acetone + MERO. Mortality rate 24 h post-exposure to eight different insecticide concentrations and using acetone + MERO as solvent. Results are the average of percentage mortalities from four to five replicates each. The bars represent the standard error on the mean (SEM).

For acetamiprid, 24 h after exposure, the recorded percentages of mortality ranged from 42.55% ± 7.02 for 0.25 µg/mL, 60% ± 9.64 for 0.5 µg/mL, 96.0% ± 2.31 for 1 µg/mL, 97.96% ± 1.18 for 2 µg/mL, 96.0% ± 1.63 for 3 µg/mL, and 100% for 5 µg/mL and above (Figure 7). This indicates that the dose of 4 µg/mL is suitable for assessing suitable for assessing malaria vectors’ susceptibility profile to acetamiprid when using acetone + MERO as solvent.

### 3.6. Assessing Possible Cross-Resistance between Clothianidin and Pyrethroids Using Genotyping of Resistance Markers

**Figure 8:**
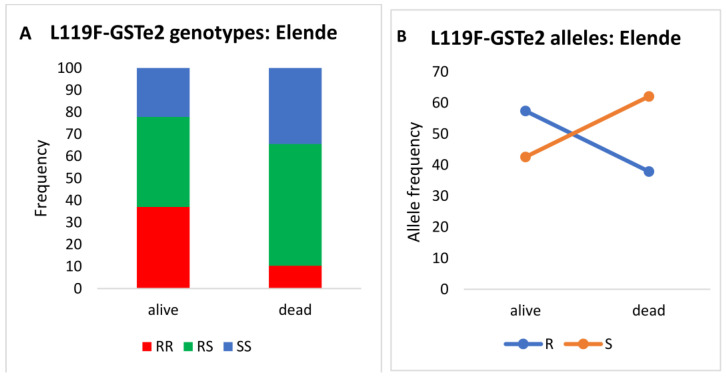
Association between the L119F-GSTe2 mutation and the resistance to clothianidin (with acetone + MERO as solvent). Distribution of genotypes (**A**) and alleles (**B**) among the dead and alive mosquitoes after exposure to clothianidin diluted in acetone + MERO. R: 119F-resistant allele and S: L119-susceptible allele.

The distribution of the L119F-GSTe2 genotypes in mosquitoes (from Elende) alive after exposure was 37.03% (10/27) for homozygous resistant (119F/F), 40.74% (11/27) heterozygotes (L119F-RS) and 22.22% (6/27) homozygous susceptible (L/L119) (Figure 8A). In those that were dead, the distribution of genotypes was as follows: 10.34% (3/29) homozygous resistant (119F/F), 55.17% (16/29) heterozygotes (L119F-RS) and 34.48% (10/29) homozygous susceptible (L/L119) (Figure 8A).

The odds ratio analysis showed a significant difference in the distribution of L119F-GSTe2 genotypes between alive and dead mosquitoes with the homozygote resistant more able to survive than the susceptible mosquitoes (OR: 5.5; *p* < 0.0001), as well as compared to heterozygotes mosquitoes (OR: 4.8; *p* < 0.0001) (Table 1). Conversely, no significant difference was observed between heterozygote and susceptible mosquitoes (OR: 1.1; *p* = 0.6).

## 4. Discussion

This study evaluated the susceptibility profile of the major malaria vector, *An. funestus* to neonicotinoids across Africa using three different solvents, and determined the diagnostic doses of imidacloprid and acetamiprid using acetone + MERO as solvent. Furthermore, we assessed the association between pyrethroid resistance markers and the ability of mosquitoes to survive neonicotinoid exposure. The stars (*) represent the significance level.

### 4.1. Contrasting Susceptibility Profiles from the Three Solvents

Neonicotinoids induced very low mortality in *An*. *funestus* populations when using absolute ethanol/acetone alone as solvent whereas adding MERO significantly increased the efficacy. The 24-h post-exposure mortality has revealed low mortality of mosquitoes in almost all locations tested with clothianidin, imidacloprid and acetamiprid when using acetone/ethanol alone as solvent. We nevertheless observed increased mortality from day 1 to day 7 after exposure confirming the slow action of neonicotinoids on the nervous system of the target insects, as previously reported by several studies [22,23]. This slow action of neonicotinoids is contrary to that observed with pyrethroids which are known to have an immediate knockdown effect in vectors [22,24]. Associating MERO (81% Rapeseed oil methyl ester) with acetone and using it as a solvent, induced an immediate positive effect on the mosquitoes tested, with 100% mortality observed 24 h after exposure in all the locations. The high mortality observed when using acetone + MERO as a solvent could be explained by the properties of MERO which is an oil with emulsifying properties [25] known to increase the solubility of the insecticide in acetone, thus preventing crystallization of the insecticide as demonstrated by several studies [13,26]. At the same time, no mortality was observed when mosquitoes were exposed to solvents only, thus indicating the non-toxicity of the solvents used, including acetone + MERO. Given the differences in mortality observed with the different solvents used, mortality rates observed with ethanol and acetone do not reflect the vectors’ exact susceptibility to the insecticides tested due to crystallization issue. However, using ethanol or acetone alone might still help capture variability between populations and even detect those populations with reduced susceptibility as reported previously [13], as the MERO could mask the resistance if not used at the right concentration. Full susceptibility was noted in mosquitoes from Gounougou to all neonicotinoids tested, whatever the solvent used. This shows that that population is less likely to develop clothianidin resistance.

Regarding the results obtained with acetone + MERO, this solvent is suitable for neonicotinoids as it induced immediate high mortality of mosquitoes and need to be recommended. However, the use of neonicotinoids diluted in acetone + MERO should be used at suitable doses such as 4 µg/mL for clothianidin as reported by Tchouakui and collaborators and the recent WHO manual for monitoring insecticide resistance in mosquito vectors [13,27] to avoid masking the development of resistance in mosquitoes. The resistance observed with imidacloprid and acetamiprid when using acetone or ethanol confirms that these solvents alone are unsuitable for assessing neonicotinoids’ efficacy on *An. funestus* as reported earlier by Tchouakui et al. (2022) in *Anopheles gambiae* populations [13] and Chouaïbou and collaborators in Côte d’Ivoire (using *Anopheles. coluzzii*) [25].

As the previous authors showed in their studies [13,28], acetone + MERO appears to be the best solvent for the commonly used neonicotinoids (clothianidin, imidacloprid and acetamiprid) and could help to better capture the accuracy of the resistance profile of a given mosquito population. However, using that solvent with the dose of insecticides used with acetone or ethanol alone could overestimate the susceptibility of mosquitoes to such insecticides. In that logic, the WHO determined the diagnostic dose of clothianidin diluted in acetone + MERO as 4 µg/mL [19]. In this study, we evaluated the diagnostic doses of imidacloprid and acetamiprid with acetone and MERO and we found that 6 µg/mL and 4 µg/mL, respectively, could be used as diagnostic concentrations for these neonicotinoids.

### 4.2. Synergist Assays Revealed That Metabolic Enzymes Are Associated with Reduced Susceptibility to Clothianidin in An. funestus

Synergist assays with PBO, DEM and DEF revealed a significant recovery of the susceptibility of mosquitoes from Elende, Mibellon and Mayuge, showing the possible implication of monooxygenases, GSTs and esterases in reduced susceptibility to clothianidin in those localities. The implication of P450s in the resistance to clothianidin has already been proved by many other authors [13,29,30,31]. The similar implication was also found in the resistance to imidacloprid by different authors in the aphids *Mirzus persicae* and *Metopolophium dirhodum* [32,33], in the Brown Planthopper *Nilavarpata lugens* [34] and in the house fly *Musca domestica* L. [35]. For the first time, we observed a positive association between the L119F-GSTe2 allele and the resistance to clothianidin diluted in acetone + MERO in *An. funestus* as previously also noticed for the I114T-GSTe2 mutation in *An. gambiae* [13], confirming the contribution of GSTs in reduced susceptibility to clothianidin. The latter represents one of the broad-spectrum enzyme families involved in the detoxification of insecticides [30]. They act by increasing the metabolic activity of insecticides, including decreasing the amount of insecticide reaching the target, thus increasing the tolerance of the insect. Moreover, the partial association between the L119F-GSTe2 mutation and the resistance to clothianidin suggests that other alleles might play a role in resistance to this insecticide.

## 5. Conclusions

The study revealed a broad susceptibility of *Anopheles funestus* populations to neonicotinoids across Africa suggesting that this insecticide class would be suitable for implementing indoor residual spraying against this species. However, the possible cross-resistance observed with the 119F-GSTe2 suggests that the selection of resistance against neonicotinoids could occur in the field population either through existing resistance mechanisms or new ones. This calls for regular monitoring of the resistance profile in Africa to ensure early detection of resistance for a suitable resistance management plan to be implemented.

## Figures and Tables

**Table 1:** Assessment of the association between L119F-GSTe2 genotypes/alleles and the ability of mosquitoes to survive clothianidin exposure. SS: susceptible; RR: homozygote resistant; RS: heterozygote; (*) significant difference.

Genotypes	L119F-GSTe2 and Clothianidin Resistance	
	Odds-Ratio	*p*-Value
**RR vs. SS**	5.55	*p* < 0.0001 **
**RR vs. RS**	4.84	*p* < 0.0001 **
**RS vs. SS**	1.14	*p* = 0.6891
**R vs. S**	2.2	*p* = 0.0058 *

## Data Availability

All data generated or analysed during this study are included within the article and its additional files.
